# EEG Alpha and Beta Band Functional Connectivity and Network Structure Mark Hub Overload in Mild Cognitive Impairment During Memory Maintenance

**DOI:** 10.3389/fnagi.2021.680200

**Published:** 2021-10-07

**Authors:** Zsuzsanna Fodor, András Horváth, Zoltán Hidasi, Alida A. Gouw, Cornelis J. Stam, Gábor Csukly

**Affiliations:** ^1^Department of Psychiatry and Psychotherapy, Semmelweis University, Budapest, Hungary; ^2^Department of Neurology, National Institute of Clinical Neurosciences, Budapest, Hungary; ^3^Department of Clinical Neurophysiology, Amsterdam Neuroscience, Vrije Universiteit Amsterdam, Amsterdam University Medical Centers, Amsterdam, Netherlands; ^4^Department of Neurology, Alzheimer Center Amsterdam, Amsterdam Neuroscience, Vrije Universiteit Amsterdam, Amsterdam University Medical Centers, Amsterdam, Netherlands

**Keywords:** mild cognitive impairment (MCI), electroencephalography (EEG), working memory (WM), functional connectivity, functional networks, minimum spanning tree (MST)

## Abstract

**Background:** While decreased alpha and beta-band functional connectivity (FC) and changes in network topology have been reported in Alzheimer’s disease, it is not yet entirely known whether these differences can mark cognitive decline in the early stages of the disease. Our study aimed to analyze electroencephalography (EEG) FC and network differences in the alpha and beta frequency band during visuospatial memory maintenance between Mild Cognitive Impairment (MCI) patients and healthy elderly with subjective memory complaints.

**Methods:** Functional connectivity and network structure of 17 MCI patients and 20 control participants were studied with 128-channel EEG during a visuospatial memory task with varying memory load. FC between EEG channels was measured by amplitude envelope correlation with leakage correction (AEC-c), while network analysis was performed by applying the Minimum Spanning Tree (MST) approach, which reconstructs the critical backbone of the original network.

**Results:** Memory load (increasing number of to-be-learned items) enhanced the mean AEC-c in the control group in both frequency bands. In contrast to that, after an initial increase, the MCI group showed significantly (*p* < 0.05) diminished FC in the alpha band in the highest memory load condition, while in the beta band this modulation was absent. Moreover, mean alpha and beta AEC-c correlated significantly with the size of medial temporal lobe structures in the entire sample. The network analysis revealed increased maximum degree, betweenness centrality, and degree divergence, and decreased diameter and eccentricity in the MCI group compared to the control group in both frequency bands independently of the memory load. This suggests a rerouted network in the MCI group with a more centralized topology and a more unequal traffic load distribution.

**Conclusion:** Alpha- and beta-band FC measured by AEC-c correlates with cognitive load-related modulation, with subtle medial temporal lobe atrophy, and with the disruption of hippocampal fiber integrity in the earliest stages of cognitive decline. The more integrated network topology of the MCI group is in line with the “hub overload and failure” framework and might be part of a compensatory mechanism or a consequence of neural disinhibition.

## Introduction

Deteriorated working memory maintenance and the impairment of visuospatial memory are early symptoms of Mild Cognitive Impairment (MCI) and Alzheimer’s disease (AD) (Bird et al., 2010; Parra et al., 2010; Gillis et al., 2013; Moodley et al., 2015) and can serve as a sensitive marker of early cognitive decline (Tierney et al., 1996; Sano et al., 2011). Visuospatial memory tests, such as the Paired Associates Learning (PAL) test are considered especially effective in the early diagnosis of MCI (Sirály et al., 2013) and in the prediction of a higher risk of developing dementia in later life (Blackwell et al., 2004).

Cognitive functions arise from the interactions between functionally connected regions of the brain (Rubinov and Sporns, 2010; Park and Friston, 2013; Stam, 2014). However, besides sufficient connections, proper cognitive functioning relies on an optimal organization of brain network (Bullmore and Sporns, 2009) and the coordinated interaction of local information processing (“segregation”) and the long-range integration of this information (Sporns, 2013; Stam, 2014). A growing body of evidence suggests that healthy brain networks are cost-efficient small-world networks combining strong local connectivity with efficient long-distance connections (Bullmore and Sporns, 2012). Furthermore, it has been shown that brain network efficiency is related to cognitive performance (van den Heuvel et al., 2009) and network measures derived from electrophysiological data can discriminate cortical network features in healthy brain and neurodegenerative brain aging (Miraglia et al., 2017; Vecchio et al., 2017).

The pathological process of AD initially affects synaptic transmission with an overall disconnection (Delbeuck et al., 2003), which could be assessed using a network approach as the structural components of the brain form a complex network at different spatial scale (from neurons to anatomical regions) from which functional dynamics arise (Vecchio et al., 2017). The abnormal functional brain network in AD has been characterized by a loss of efficiency, disturbed community structure, and selective hub vulnerability in both structural and functional network studies (Tijms et al., 2013; Stam, 2014; Miraglia et al., 2017). Furthermore, the extent of network changes correlates with the extent of the underlying structural pathology, with the severity of the clinical symptoms, and with disease duration (Stam, 2014).

There is an increasing demand for functional markers of early cognitive decline to identify patient populations that have an increased risk of developing dementia as these individuals are the best applicants for therapeutic intervention. Previous EEG studies revealed potential spectral and functional connectivity (FC) biomarkers that are able to predict the future progression of cognitive decline (Moretti et al., 2011; Toth et al., 2014; Mazaheri et al., 2018; Sharma et al., 2019).

The assessment of functional connectivity and network topology can provide an integrative approach that can reflect progressive brain dysfunction in MCI and AD (Pievani et al., 2011; Stam, 2014; Hallett et al., 2020). Moreover, graph theory approach could provide a general language that could help us to understand how cortical atrophy and functional disruptions are linked together in the pathological processes of AD (Bullmore and Sporns, 2009; Stam, 2014; Miraglia et al., 2017; Douw et al., 2019) and to discover novel early diagnostic and predictive neurophysiological markers (Rossini et al., 2016; Horvath et al., 2018).

There is a considerable amount of literature reporting decreased resting-state functional connectivity in MCI and AD in the alpha- and beta frequency range (Stam et al., 2003; Stam, 2014; Babiloni et al., 2016; Koelewijn et al., 2017; Horvath et al., 2018; Núñez et al., 2019; Briels et al., 2020). Changes in memory task-related functional connectivity are much less investigated and former studies reported mixed results (Hogan et al., 2003; Pijnenburg et al., 2004; Jiang and Zheng, 2006; Hou et al., 2018). The conflicting results might be partly explained by differences in the diagnostic criteria of the study groups (clinical or biomarker-based, MCI or AD patients), sample size, and the choice of functional connectivity measure, some of which are not corrected for the effect of volume conduction, which might influence previous results (de Waal et al., 2014; Herreras, 2016).

Regarding the overall network structure, previous studies observed a progressive derangement of brain organization during the disease course causing a deviation from the optimal small-world architecture to a more random type configuration leading to a less efficient information transfer during resting state (de Haan et al., 2009; Stam et al., 2009; Stam, 2014; Wei et al., 2015; Miraglia et al., 2017), and cognitive tasks (Wei et al., 2015; Das and Puthankattil, 2020), firstly affecting alpha-band networks in MCI (Miraglia et al., 2017).

Former studies highlighted the role of hubs in network disturbances in MCI and AD (Stam, 2014), which are nodes with high values of relative importance—such as node degree or betweenness centrality—and take a central role in network organization by facilitating the optimal flow within healthy brain networks (van den Heuvel and Sporns, 2013; Stam, 2014). Hub regions have been found especially vulnerable in AD (Stam et al., 2009; D’Amelio and Rossini, 2012; de Haan et al., 2012; Tijms et al., 2013; Crossley et al., 2014; Stam, 2014; Miraglia et al., 2017; Yu et al., 2017) and disruption of the global network structure in AD has been explained by the overload and failure of hub nodes (de Haan et al., 2012; Stam, 2014). Throughout the disease progression neural activity, functional connectivity, and hub activity follow an inverted U shape: increasing in early MCI, followed by a decrease in late MCI and AD (de Haan et al., 2012).

From a network perspective, visuospatial memory in MCI is an area of particular interest, as neuronal networks associated with this cognitive function are particularly affected by the neuropathological process of AD (Pievani et al., 2011), especially frontoparietal and frontotemporal connections (Babiloni et al., 2016). Moreover, using memory tasks enhances EEG abnormalities related to MCI and improves the classification accuracy of healthy subjects and patients (van der Hiele et al., 2007a; San-Martin et al., 2021). Therefore we applied a computerized implementation of a visuospatial memory task in the current study.

Our study aimed to analyze EEG functional connectivity and network differences in the alpha and beta frequency band during memory maintenance between MCI patients and healthy elderly with subjective memory complaints.

Former studies reported decreased alpha and beta-band AEC-c in AD (Koelewijn et al., 2017; Núñez et al., 2019; Briels et al., 2020), therefore we hypothesized a decreased alpha- and beta-band functional connectivity in MCI patients and we expected that the memory load-related modulation of global functional connectivity will be less prominent in the MCI patients than the control subjects, since their reduced available cognitive capacity.

In accordance with the early increase of network integration suggested by the “hub overload and failure” framework (Stam, 2014) and based on previous MST network studies (Engels et al., 2015; Lopez et al., 2017; Wang et al., 2018) we hypothesized a more centralized network topology in MCI patients. As hub nodes are exposed to an increased traffic load in a more centralized network, this transition might lead to the overload and subsequent failure of these hub nodes and the disturbance of the modular system of the network (Stam, 2014). Therefore, the shift to a more integrated network configuration might reflect the increased vulnerability of brain networks in MCI.

## Materials and Methods

### Participants and Clinical Measures

The study was carried out in the Department of Psychiatry and Psychotherapy, Semmelweis University, Budapest, Hungary. EEG was recorded from 17 MCI patients and 20 healthy control participants during a visuospatial memory task. Among them, structural MRI data of 13 MCI patient and 13 control participant and diffusion-weighted MRI (DW-MRI) data of 10 MCI patient and 17 control participant was available (10 MCI patient and 13 healthy control subject had both structural and functional MRI data). Participants had subjective memory complaints and applied to take part in a cognitive training program announced among general practitioners and in a Retirement Home (The study is registered at ClinicalTrials.gov, the identifier is “NCT02310620”). Every participant underwent a regular psychiatric assessment to evaluate possible excluding comorbidity. After that, cognitive functions were assessed with neuropsychological tests to specify the diagnosis [Addenbrooke’s Cognitive Examination (ACE), Rey Auditory Verbal Learning Test (RAVLT), Trail Making Test (TMT)]. Participants were not financially compensated for their participation but received a detailed written feedback on their performance on the neuropsychological tests.

The diagnostic procedure of MCI was based on the Petersen criteria (Petersen, 2004), including subjective memory complaints corroborated by an informant, preserved everyday activities, memory impairment based on a standard neuropsychological test, preserved global cognitive functions, and the exclusion of dementia. For the detailed assessment of memory impairment, we applied the Rey Auditory Verbal Learning Test (RAVLT) (Strauss et al., 2006). Attention, executive functions, and cognitive flexibility were examined with the Trail Making Test (TMT) Part A and Part B (Tombaugh, 2004; Strauss et al., 2006), global cognitive performance was estimated with the Addenbrooke’s Cognitive Examination (ACE) (Mathuranath et al., 2000). For the differentiation between MCI and healthy controls, we applied a cut-off score of 1 SD under population mean standardized for age and gender/education in these neuropsychological tests. Participants, who scored under the cut-off value in the delayed recall subscore or the total score of RAVLT or the TMT Part B or the ACE, were put into the MCI group. Subjects with dementia were excluded based on cognitive impairment according to the Mini-Mental State Examination (MMSE) scores standardized for age and education (Strauss et al., 2006) and on the loss of ability to perform activities of daily living. The Geriatric Depression Scale (GDS) was used to assess depressive symptoms (Yesavage, 1988), while anxiety symptoms were measured by the Spielberger State-Trait Anxiety Inventory (STAI) (Spielberger et al., 1970). Exclusion criteria were history of head trauma with loss of consciousness, prior CNS infection, epileptic seizure, clinically significant brain lesions (stroke, severe periventricular white matter disease, clinically significant white matter infarcts), multiple sclerosis or other demyelinating disorders, hydrocephalus, untreated vitamin B12 deficiency, untreated hypothyroidism, syphilis or HIV infection, mental retardation, major depression, schizophrenia, other acute psychiatric disorder, electroconvulsive therapy, renal insufficiency, liver disease, significant systemic medical illness, alcohol, or substance use dependency. Demographic and neuropsychological data are summarized in Table 1.

**TABLE 1:** Demographic data and results of basic neuropsychological tests.

	**control (*n* = 20)**	**MCI (*n* = 17)**	***p*-value**
Age [Mean (SD)]	65.2 (6.9)	69.9 (6.5)	*p* = 0.04
Education^a^	15%/15%/70%	18%/18%/65%	n.s.*
Gender (female)	70%	41.2%	n.s.*
Rey Auditory Verbal Learning Test 1–5 sum^b^	54.3 (7.8)	40.0 (11.3)	*p* < 0.0001
Rey Auditory Verbal Learning Test delayed recall^c^ (MCI: *n* = 13)	11.4 (2.6)	7.2 (4.4)	*p* = 0.007
ACE total score^d^	94.9 (2.9)	86.2 (8.3)	*p* = 0.0006
ACE VL/OM-ratio^e^	2.6 (0.3)	2.8 (0.6)	n.s.*
Mini mental state examination total score^f^	29 (1.2)	27.9 (1.4)	*p* = 0.02
Trail Making Test Part A^g^	34.9 (10.8)	70.6 (52.9)	*p* = 0.006
Trail Making Test Part B^g^ (MCI: *n* = 16)	69.0 (22.7)	143.5 (69.2)	*p* < 0.0001
Geriatric Depression Scale score^h^ (Control: *n* = 19)	3.6 (2.9)	4.3 (3.5)	n.s.*
STAI score^i^	39.4 (11.0)	35.8 (9.4)	n.s.*

*MCI, Mild cognitive impairment; ACE, Addenbrooke’s cognitive examination; STAI, State-trait anxiety inventory.*

*^a^Participants were categorized into three education groups: 1 = less than 12 years; 2 = high school graduation (12 years education); 3 = more than 12 years of education.*

*^b^Sum of all words in the first five trials. The maximum score is 75.*

*^c^The maximum score is 15.*

*^d^The maximum score is 100.*

*^e^VL/OM: verbal fluency and language points/orientation and delayed recall ratio can be defined based on ACE. A result below 2.2 indicate frontotemporal dementia and a result over 3.2 indicate Alzheimer’s disease.*

*^f^The maximum score is 30.*

*^g^Time needed for completing the task in seconds.*

*^h^The maximum score is 15.*

*^i^The maximum score is 80.*

*n.s. (not significant) = *p* > 0.05.

### EEG Paradigm and Procedures

EEG examinations were carried out on weekdays between 10 a.m. and 4 p.m. Participants were seated in a dimly lit, sound-attenuated room. All participants had normal or corrected-to-normal vision.

**FIGURE 1:**
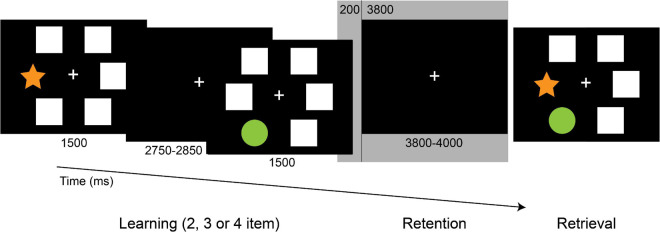
The Paired Associates Learning (PAL) task. Following the memory sequence, participants indicated, whether the shapes appeared in the previously presented positions. Epochs of 4,000 ms duration of the retention period (from 200 ms pre-stimulus to 3,800 ms post-stimulus, highlighted) were included in the analysis.

To measure visuospatial memory, during the EEG recording participants performed an implementation of the PAL test used in several neuropsychological test batteries (Sirály et al., 2013). White windows and colored shapes sized 2.65 cm × 2.65 cm were presented as stimuli on a computer screen at approximately 50 cm distance with Presentation 13.0 software (Neurobehavioral Systems, Inc.; Albany, CA). At the onset of each trial, eight blank windows appeared on the screen for 1,500 ms. After that, two, three, or four random windows opened up sequentially for 1,500 ms with abstract shapes shown in them, separated by a fixation cross for 450–500 ms. Meanwhile, other windows remain blank depending on the difficulty level. For the retention period, a fixation cross appeared for 3,800–4,000 ms. During the retrieval period, the previously shown shapes reappeared in the windows, and participants were instructed to indicate by clicking with the mouse (yes-right/no-left) whether the shapes popped up in the same positions they saw them before (Figure 1). The test consisted of 72 trials in total (32 two-item, 24 three-item, 16 four-item). The response assignment was counterbalanced across trials. Efficiency was measured by response accuracy.

It was carefully monitored that the participants understood the instructions and stayed alert during the session to bypass the possible distorting effect of extended eye closure on the EEG activity, especially in the alpha frequency range (Barry et al., 2007). For the same purpose, participants completed the task in three parts separated by a 3-min rest period.

### EEG Recording and Processing

EEG was recorded from DC with a low-pass filter at 100 Hz using a high-density 128-channel BioSemi ActiveTwo amplifier (Metting van Rijn et al., 1990). Electrode caps had an equidistant layout and covered the whole head according to the Biosemi equiradial montage. Eye movements were monitored with EOG electrodes placed below the left and above the right external canthi. Data were digitized at a sampling rate of 1,024 Hz. Built-in and self-developed functions as well as the freeware EEGLAB toolbox (Delorme and Makeig, 2004) in the Matlab (MathWorks, Natick, MA) development environment was used for subsequent off-line data analyses. EEG was re-referenced to the common average reference and filtered off-line between 0.5 and 45 Hz using zero-phase shift forward, and reverse IIR Butterworth filter. As four channels (P2, FT7h, P7, P9) were exceptionally noisy across multiple subjects, they were removed from the recordings for all subjects prior to the analysis. Epochs from 500 ms pre-stimulus to 4,500 ms post-stimulus for the retention period were extracted from the continuous EEG. Removal of muscle, blinking, and eye movement artifacts (detected by EOG) were performed by the Multiple Artifact Rejection Algorithm (MARA), a machine-learning algorithm that evaluates the ICA- (Independent Component Analysis) derived components (Winkler et al., 2011, 2014), Furthermore, epochs with a voltage exceeding ±100 μV on any channel were rejected from the analysis. After artifact rejection, the average number of trials in the control group and in the MCI group were 71.7 (*SD* = 0.7) and 71.1 (*SD* = 2.3) for the retention condition, respectively.

### EEG Data Analysis

After artifact rejection, epochs of 4,000 ms duration of the retention period (from 200 ms pre-stimulus to 3,800 ms post-stimulus, sampling rate 1,024 Hz, 4,096 time points) were extracted from the EEG recording, which, based on previous studies, we assumed to be sufficient to measure oscillatory activity in the alpha and beta frequency band (Fraschini et al., 2016). EEG connectivity analyses were performed with open-access software BrainWave (version 0.9.152.12.26; available at http://home.kpn.nl/stam7883/brainwave.html). Functional connectivity between EEG channels was analyzed by measuring the amplitude envelope correlation with leakage correction (AEC-c) calculated for all EEG epochs of each subject, after having band-pass filtered the EEG time-series in the alpha (8–13 Hz) and beta (13–30 Hz) frequency band. The amplitude envelope correlation (AEC) measures the linear correlations of the envelopes of the band-pass filtered and Hilbert-transformed signals (Bruns et al., 2000). The leakage-corrected version of the AEC (Hipp et al., 2012) uses a pair-wise symmetric orthogonalization procedure before the calculations of the AEC to remove zero-lag correlation correlations that could be attributed to spurious connectivity caused by volume conduction. Therefore, it is considered a reliable measure of genuine functional connectivity (Brookes et al., 2011; Hipp et al., 2012; Colclough et al., 2016; Briels et al., 2020). Connectivity metrics were averaged over epochs creating values for each electrode at the patient level. Global functional connectivity values were calculated by averaging the AEC-c of all electrodes.

We carried out a spectral analysis to assess whether the detected effects were solely driven by differences in spectral power or peak frequency. Relative power in alpha and beta frequency band and peak frequency (Hz; dominant frequency between 4 and 13 Hz) were calculated with the BrainWave software using Fast Fourier Transformation.

### Graph-Theoretical Analysis

The graph-theoretical representation of the functional connectivity matrix was constructed by the Minimum Spanning Tree (MST), which is a simplified representation of the core network containing the strongest and most relevant “backbone” connections (Stam et al., 2014; Tewarie et al., 2015) that can reflect topological changes (Tewarie et al., 2015). Former studies pointed out that graph theoretical measures are dependent on network size and density, which can make the comparison across different groups and conditions by using conventional network analytical methods challenging (van Wijk et al., 2010; Fornito et al., 2013; Stam et al., 2014). The MST calculation overcomes the bias of network density and degree without any additional normalization step by forming an acyclic subnetwork using the strongest available connections without forming loops and connecting all nodes with a fixed number [(number of nodes) - 1) of edges. MST graphs were generated for each participant, epoch for alpha and beta frequency band, based on the full connectivity matrix constructed from the AEC-c values obtained for each pair of electrodes. MST metrics were averaged over epochs for each subject.

**FIGURE 2:**
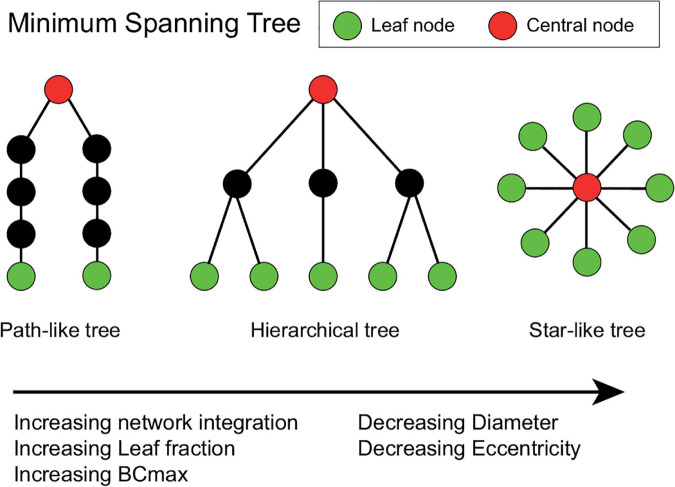
Schematic representation of three minimum spanning trees (MSTs). MST structures can range from a path-like tree (i.e., minimally integrated network) to a star-like tree (i.e., maximally integrated network). Green nodes represent leaf nodes (i.e., end-nodes in the graph), while red nodes represent central nodes. The hierarchical tree combines the relatively small diameter with the relatively low betweenness centrality (BCmax) value, which prevents information overload on the central node making this an optimal configuration (Stam and van Straaten, 2012). The Figure was adjusted from van Dellen et al. (2014) and van Lutterveld et al. (2017).

Two extreme topologies of MST can be distinguished: a path-like and a star-like shape. In a path, all nodes are linked to exactly two other nodes, except the two nodes at the extremities of the tree. These nodes are connected to only one other node and are referred to as the leaves of the tree. In the case of a star shape, all but one node are linked to a central node (Stam et al., 2014). Between these two shapes, MST-s can have various configurations (Figure 2).

The diameter of the tree is the maximum number of edges between any two nodes of the network. Leaf fraction is the number of nodes with exactly one connection divided by the total number of nodes of the tree. Degree refers to the number of edges connected to a node. Betweenness centrality (BC) of a node refers to the normalized fraction of all paths connecting two nodes that pass through the selected node, and it characterizes the “hubness” of the node within the network. The eccentricity of a node denotes the longest shortest path to any other node in the MST. Degree divergence (kappa—κ) measures the broadness of the degree distribution, which shows high value in networks with high-degree hubs, and it is related to the resilience of the network against attacks. In an MST the most efficient communication can be achieved in a star-like configuration, as it has the shortest possible average path length between two arbitrary nodes, however, in this case, the central node might easily be overloaded. This trade-off between large-scale integration and the overload of central nodes is quantified by the tree hierarchy. The optimal MST topology balances efficiency and node load.

Global and node-specific parameters were computed with the Brainwave software, based on the measures described by previous studies (Stam et al., 2014; Tewarie et al., 2015), summarized in Table 2. Degree, betweenness centrality, and eccentricity were calculated for each node separately, and the maximum degree, maximum BC, and mean eccentricity were included in the statistical analysis as global characteristics of the MST. Global MST network parameters were averaged across epochs.

**TABLE 2:** Explanation of concepts and terminology based on Tewarie et al. (2015) and Van Dellen et al. (2015).

**Measure**	**Explanation**	**Formula**
Nodes (N)	Number of nodes	
Links (M)	Number of links/maximum leaf number	
Degree (k)	Number of links for a given node. Nodes with a high degree may be considered hubs. We used the maximum degree to characterize the strength of the most important node of the network.	$k_{i}=\sum_{j\in N}a_{i\,j}$
Leaf fraction (L_*f*_)	Fraction of leaf nodes (L) in the MST where a leaf node is defined as a node with only one connection. It describes to what extent the network has a central organization. A high leaf fraction indicates, that communication is largely dependent on hub nodes.	*L*_*f*_=*L*/*M*
Diameter	Longest distance between any two nodes in an MST, normalized by the total number of connections. In a network with a low diameter, information is efficiently processed between remote brain regions. The diameter is also related to the leaf number: the value of the diameter decreases when the leaf number increases.	*D*=*d*/*M*
Eccentricity	Longest shortest path from a reference node to any other node in the MST. Eccentricity is low if the node is located in the center of the tree. Eccentricity of the network describes how efficient information is communicated from the least central node.	
Betweenness centrality (BC)	Fraction of all shortest paths that pass through a particular node. BC ranges between 0 (leaf node) and 1 (central node in a star-like network). Nodes with a high BC are considered hub nodes based on their importance for global communication in the network. The BC of the tree was characterized by the maximum BC value, which describes the importance of the most central node and it is a measure of central network organization.	$B\,C_{i}=\frac{1}{\left(n-1\right)\,\left(n-2\right)}\,\sum_{\begin{array}{c} h,j\in N\\ h\,\neq \,ȷ,h\,\neq \,i,\neq \,i \end{array}}\frac{\rho_{h\,j}^{\left(i\right)}}{\rho_{h\,j}}\,\,$ ρih is the number of shortest paths between h and j, and ρih(i) is the number of shortest paths between h and j that pass through i
Degree divergence (*κ*)	Measure of the broadness of the degree distribution. Related to resilience against attacks, epidemic spreading and the synchronizability of complex networks	$\kappa =\frac{\left\langle k^{2}\right\rangle }{\left\langle k\right\rangle }$
Tree hierarchy (T_*H*_)	Quantifies the trade-off between large scale integration in the MST and the overload of central nodes It characterizes the hypothesized optimal topology of brain network organization, where information is transferred between brain regions in the fewest possible steps, while preventing information overload of central brain regions.	$T_{H}=\frac{L}{2\,M\,B\,C_{m\,a\,x}}$

### MR Image Acquisition and Processing and Diffusion Tensor Fitting

The obtained structural gray matter volumetric (cortical thickness and subcortical brain structure volumes) and the diffusion-weighted data were previously published by our study group (Csukly et al., 2016; Gyebnár et al., 2018).

Participants underwent a routine brain MR examination, producing high-resolution anatomical images used for analysis. Image acquisitions were made at the MR Research Center, Semmelweis University, Budapest on a 3 Tesla Philips Achieva clinical MRI scanner equipped with an 8-channel SENSE head coil. High resolution, whole-brain anatomical images were obtained using a T1 weighted 3-dimensional spoiled gradient echo (T1W 3D TFE) sequence. 180 contiguous slices were acquired from each subject with the following imaging parameters: *TR* = 9.7 ms; *TE* = 4.6 ms; flip angle = 8°; FOV of 240 mm × 240 mm; voxel size of 1.0 × 1.0 × 1.0 mm. Brain DW-MRI images were collected with a single shot SE-EPI sequence, with *b* = 800 s/mm^2^ diffusion weighting in 32 directions and one *b* = 0 image. In-plane resolution was 1.67 × 1.67 mm; whole-brain coverage was achieved with 70 consecutive, 2 mm thick axial slices; *TR* = 9,660 ms repetition time, *TE* = 75.6 ms echo time, and 90° flip angle was used; the total acquisition time was 8:32 min.

Cortical reconstruction, volumetric segmentation and parcellation of the MRI data into standardized region of interest (ROIs) were performed automatically by Freesurfer 5.3 image analysis suite^1^ (see details in Csukly et al., 2016), however, segmentation and cortical models were checked and corrected manually on each subject. Volumetric measurements were normalized by dividing by the intracranial volume (ICV) also computed during the Freesurfer pipeline, while cortical thickness measurements were included in the analysis without further normalization based on previous results (Westman et al., 2013).

DWI data were preprocessed using the Matlab-based ExploreDTI software package (Leemans et al., 2009). Processing steps included coordinate system transformation, rigid body transformations for correcting subject motion, non-rigid transformations for correcting susceptibility-related and EPI-induced distortions, with the local rotation of the b-matrix (the diffusion weighting directions) to avoid angular inaccuracies (Leemans and Jones, 2009). The high-resolution T1-weighted images were used as templates for registration to correct the distortions inherent to the EPI-acquisition method (Jezzard et al., 1998); thereby DW-images were spatially aligned to the T1W images. After tensor fitting, using the RESTORE (Robust Estimation of Tensors by Outlier Rejection) (Chang et al., 2005) algorithm, two voxel-wise DTI-measures, fractional anisotropy (FA) and mean diffusivity (MD) (Pierpaoli and Basser, 1996; Alexander et al., 2011; Basser and Pierpaoli, 2011) were calculated from the tensor eigenvalues, following their well-established definitions, to be used in voxel-level and ROI-based analyses (see Gyebnár et al., 2018 for details on tensor fitting and DTI scalar calculations).

### Statistical Analysis

Demographic characteristics, results of the neuropsychological tests, and response accuracy of the study groups were compared with independent samples *t*-tests, Mann-Whitney *U* tests, or χ^2^ tests where appropriate. Normal distribution of variables was tested using the Kolmogorov–Smirnov test.

Group comparisons of global functional connectivity and MST metrics were performed on the EEG from three levels of memory load conditions (two-item, three-item, four-item), while we used the average of these conditions for the correlational analysis with the size of medial temporal lobe structures and hippocampal fiber integrity.

Functional connectivity and network parameters of the two study groups were tested by two-way analysis of covariance (ANCOVA) of the study group (HC vs. MCI) × memory load (two- vs. three vs. four-item sequence). All the main effects including age as a covariate and two-way interactions were included in the ANCOVA model. Statistical significance was determined at *p* < 0.05.

*Post-hoc* pairwise contrasts were conducted to investigate the interactions. Since between-group comparisons were evaluated over three levels of memory load, Hochberg correction for multiple comparisons was applied to the *post-hoc* contrasts (Hochberg, 1988; Hochberg and Benjamini, 1990). To characterize the magnitude of the reported effects we reported the values of effect size (Cohen’s d) (Ferguson, 2009).

Structural and DW-MRI results were derived from previously published parts of our study (Siraly et al., 2015; Csukly et al., 2016; Gyebnár et al., 2018). We followed a ROI-based approach and assessed the association between functional connectivity and early-stage medial temporal lobe atrophy and hippocampal fiber integrity as these are important early markers of MCI (Márquez and Yassa, 2019). As the MRI results of some participants were outlier values, we applied the Spearman correlation in the analyses which is robust against the effect of outliers.

## Results

### Demographic and Neuropsychological Characteristics

In total, 17 MCI patients (mean age 69.9 ± 6.5 years; 7 females) and 20 healthy control participants (mean age 65.2 ± 6.9 years; 14 females) were included in the study. Groups did not differ with regard to gender, level of education, depressive symptoms (GDS score), and anxiety symptoms (STAI-score). However, MCI patients were older than the control participants, therefore statistical tests were corrected for age as a covariate. Furthermore, patients with MCI had a significantly lower score on the neuropsychological tests (ACE, MMSE, RAVLT, MMSE) than the control participants (Table 1).

### Behavioral Results

In the PAL task response accuracy of the MCI patients showed a trend level decrease compared to the control group (MCI: mean = 77.2% *SD* = 21.2, HC: mean 88.4 = % *SD* = 7.2, *U* = 106.5, *Z* = 1.9, *p* = 0.05, Cohen’s *d* = 0.8). The control group had a significantly lower score in the high memory load (four-item) condition compared to the low memory load (two-item) (*Z* = –3.4, *p* = 0.0006) and to the medium memory load (three-item) condition (*Z* = 2.9, *p* = 0.0041), while in the MCI group no significant memory load-related differences were observed in response accuracy.

### Functional Connectivity in the Alpha Band

**FIGURE 3:**
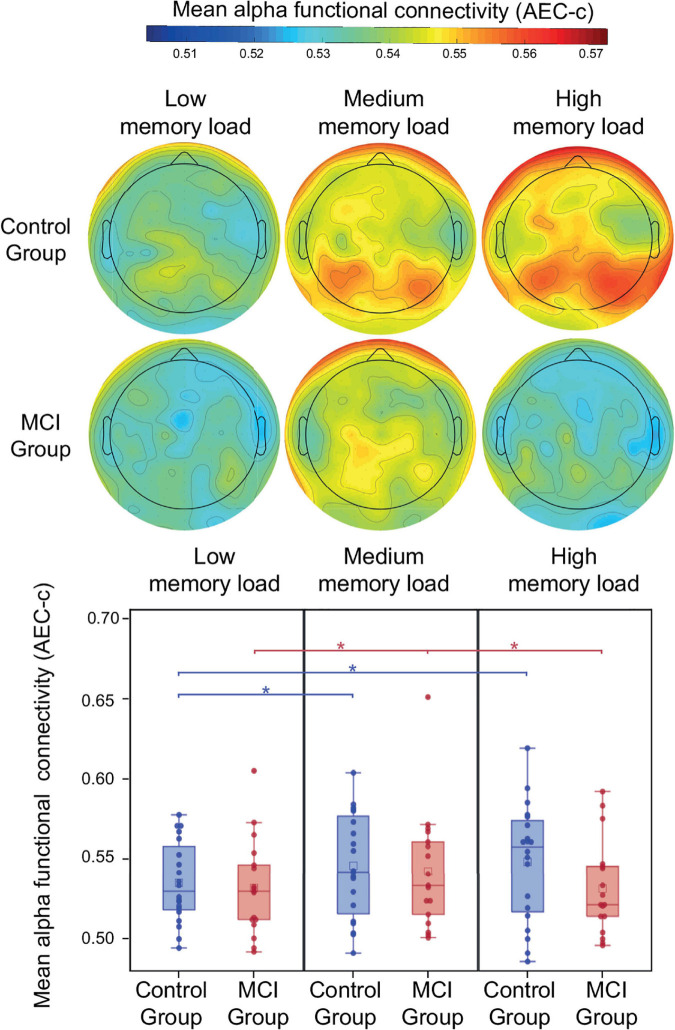
Mean alpha AEC-c in the retention condition of the PAL test. Functional connectivity in the alpha frequency band (measured by AEC-c) during the retention period of the PAL test and topographical representation of the mean AEC-c values of EEG electrodes (i.e., the average functional connectivity strength to all other electrodes). *p < 0.05.

During the retention period of the PAL test memory load had a significant modulatory effect on alpha AEC-c [*F*(2, 34) = 5.92, *p* = 0.006] (Figure 3). Furthermore, a trend-level interaction of group and memory load was observed [*F*(2, 34) = 3.03, *p* = 0.06]. The mean alpha AEC-c and the topography of average connectedness (i.e., mean AEC-c of each electrode) are shown in Figure 3. *Post-hoc* analysis of this interaction revealed, that the memory load-related modulation of AEC-c followed different dynamics in the two study groups: in the control group compared to the low memory load condition (two-item), a significantly increased mean AEC-c was observable in the medium memory load condition (three-item; *t* = 2.59, *df* = 34, *p* = 0.01, Cohen’s *d* = 0.4) and in the high memory load condition (four-item; *t* = 2.88, *df* = 34, *p* = 0.007, Cohen’s *d* = 0.4) and these memory load-related differences remained significant after correction for multiple comparisons.

In contrast to that, the MCI group showed a significantly increased mean AEC-c in the medium memory load condition compared to low memory load (*t* = 2.28, *df* = 34, *p* = 0.03, Cohen’s *d* = 0.3), while in the high memory load condition a significantly diminished mean functional connectivity was observable compared to medium memory load (*t* = 2.5, *df* = 34, *p* = 0.02, Cohen’s *d* = 0.3), however, these differences became trend level after correction for multiple comparisons (corrected *p* = 0.06 and 0.05, respectively). Study group and age did not have a significant effect on alpha functional connectivity (*p* > 0.05).

### Functional Connectivity in the Beta Band

**FIGURE 4:**
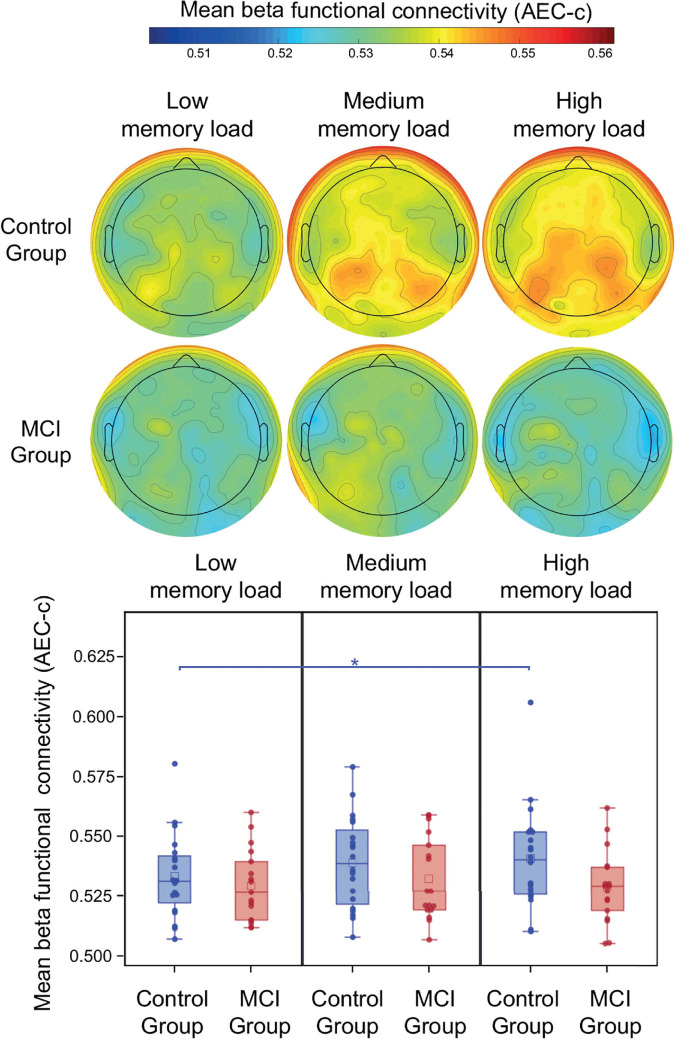
Mean beta AEC-c in the retention condition of the PAL test. Functional connectivity in the beta frequency band (measured by AEC-c) during the retention period of the PAL test and topographical representation of the mean AEC-c values of EEG electrodes (i.e., the average functional connectivity strength to all other electrodes). *p < 0.05.

During the retention period of the PAL test study group, memory load and age did not have a significant effect on beta AEC-c (*p* > 0.05). The mean beta AEC-c and the topography of average connectedness (i.e., mean AEC-c of each electrode) are shown in Figure 4. Interaction of study group and memory load was not significant, however, the *post-hoc* analysis revealed that in the control group mean beta functional connectivity in the high memory load condition was significantly increased compared to the low memory load condition (*t* = 2.82, *df* = 34, *p* = 0.008, Cohen’s *d* = 0.4), which remained significant after correction for multiple comparisons, while in the MCI group no memory load-related differences were observable (Figure 4).

### Correlational Analysis of Alpha and Beta Functional Connectivity and the Size and Fiber Integrity of the Medial Temporal Lobe Structures

**FIGURE 5:**
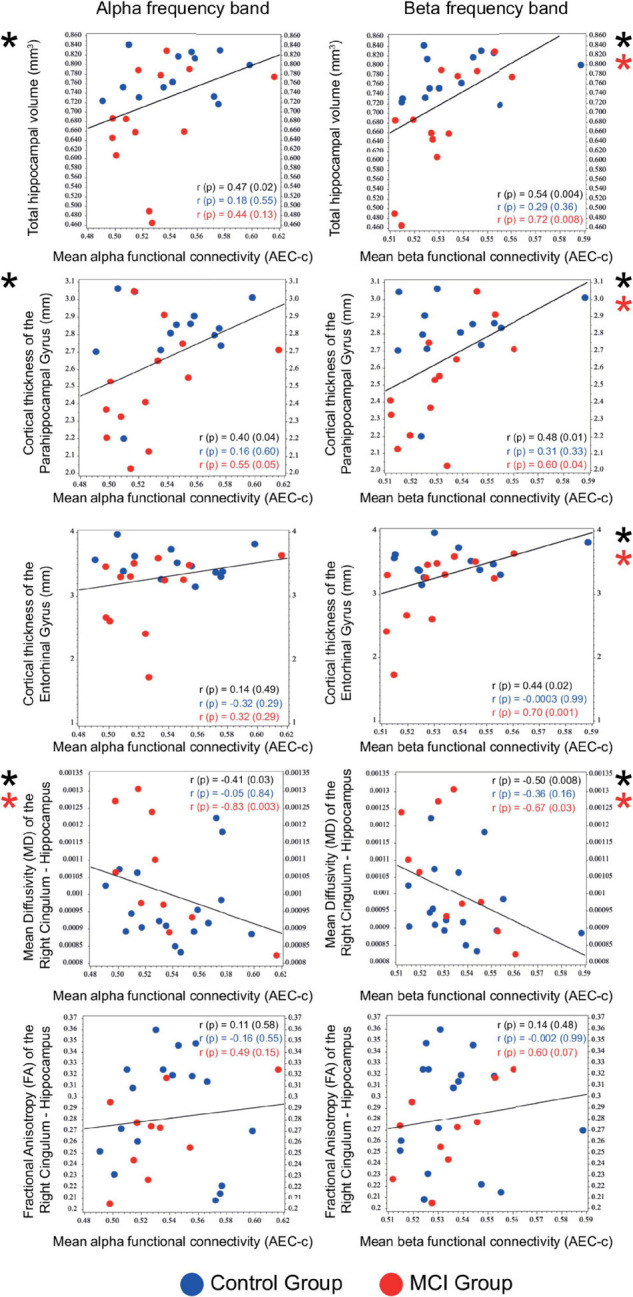
Correlation of mean functional connectivity (AEC-c) in the alpha and beta band with the size (*n* = 26) and DTI measures (*n* = 27) of medial temporal lobe structures. Spearman r and *p*-values are reported for the total sample (black) and for the two study groups (Control group: blue, MCI group: red). Significant correlations are marked with an asterisk.

Correlational analysis of mean functional connectivity averaged over all conditions and structural and DW-MRI results of medial temporal lobe structures (relative hippocampal volume, cortical thickness of the parahippocampal and the entorhinal gyrus, mean diffusivity (MD), and fractional anisotropy (FA) of the right and left cingulum—hippocampal subdivision) was performed on the entire sample. Mean alpha and beta AEC-c showed a significant positive correlation with the total relative hippocampal volume (alpha AEC-c: Spearman *r* = 0.47, *p* = 0.02, beta AEC-c: Spearman *r* = 0.54, *p* = 0.004), and with the cortical thickness of the parahippocampal gyrus (alpha AEC-c: Spearman *r* = 0.40, *p* = 0.04, beta AEC-c: Spearman *r* = 0.48, *p* = 0.01) and a significant negative correlation with the mean diffusivity of the right cingulum—hippocampal subdivision (alpha AEC-c: Spearman *r* = –0.41, *p* = 0.03, beta AEC-c: Spearman *r* = –0.50, *p* = 0.008). Furthermore, mean beta AEC-c correlated significantly with the cortical thickness of the entorhinal gyrus (beta AEC-c: Spearman *r* = 0.44, *p* = 0.02) (Figure 5).

Correlations of the mean beta AEC-c and structural MRI results were driven by the MCI group (relative hippocampal volume: Spearman *r* = 0.72, *p* = 0.008, parahippocampal gyrus Spearman *r* = 0.60, *p* = 0.04, entorhinal gyrus Spearman *r* = 0.70, *p* = 0.01). Moreover, correlations between the mean alpha and beta AEC-c and the mean diffusivity of the right hippocampal cingulum were driven by the MCI group (alpha AEC-c: Spearman *r* = –0.83, *p* = 0.003, beta AEC-c: Spearman *r* = –0.67, *p* = 0.03). Detailed results of the correlational analysis with stratified by diagnosis can be found in Supplementary Table 1.

### Spectral Analysis

Our results showed that while study group [*F*(1, 34) = 0.02, *p* = 0.88] and age [*F*(1, 34) = 1.07, *p* = 0.30] did not have a significant effect on relative alpha power, memory load had a modulatory effect on relative alpha power [*F*(2, 34) = 4.04, *p* = 0.03]. Interaction of study group and memory load showed a trend level effect [*F*(2, 34) = 3.13, *p* = 0.06]. The *post-hoc* analysis revealed that in the control group the relative alpha power in the high memory load condition was significantly increased compared to the low memory load condition (*t* = 3.69, *df* = 34, *p* = 0.0006, Cohen’s *d* = 0.3), which remained significant after correction for multiple comparisons.

In the beta band neither study group [*F*(1, 34) = 1.26, *p* = 0.27] nor memory load [*F*(2, 34) = 0.41, *p* = 0.67] or age [*F*(1, 34) = 0.90, *p* = 0.35] had a significant effect on relative beta power. Interaction of study group and memory load showed a trend level effect [*F*(2, 34) = 0.06, *p* = 0.94]. The *post-hoc* analysis revealed no significant effects.

Furthermore, study groups did not have a significantly different peak frequency [*F*(1, 34) = 0.21, *p* = 0.65], however memory load had a significant modulatory effect on the peak frequency values [*F*(2, 34) = 6.44, *p* = 0.043]. Interaction of group and memory load showed a trend level effect [*F*(2, 34) = 2.97, *p* = 0.07], however, the *post-hoc* analysis revealed that in the control group the peak frequency in the high memory load condition was significantly increased compared to the low memory load condition (*t* = 3.87, *df* = 34, *p* = 0.0005, Cohen’s *d* = 0.3), which remained significant after correction for multiple comparisons. Furthermore, there was no significant difference between the two study groups regarding the mean peak frequency values (averaged over memory loads) (MCI: mean = 8.5 Hz, *SD* = 1.4, HC: mean = 8.2 Hz, *SD* = 1.3, *t* = 0.75, *df* = 35, *p* = 0.46). Distribution of mean relative power in the alpha and beta frequency band and peak frequency by study groups can be found in Supplementary Figure 1.

### Minimum Spanning Tree Parameters in the Alpha Band

The network analysis (calculated over all memory load conditions) indicated that the MCI group had a significantly decreased MST diameter compared to the control group [*F*(1, 34) = 5.36, *p* = 0.03]. Furthermore, a decreased eccentricity was observed in the MCI group [*F*(1, 34) = 4.85, *p* = 0.03]. However, age also had a significant mean effect on these parameters [*F*(1, 34) = 4.64, *p* = 0.04 and *F*(1, 34) = 4.14, *p* = 0.05, respectively].

**FIGURE 6:**
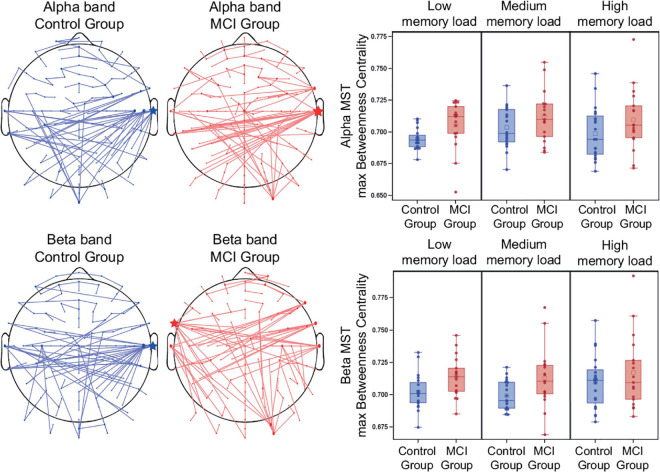
Group MSTs and maximum Betweenness Centrality in the alpha and beta frequency band. Group-average MSTs of the two study groups (average of all levels of memory load). The size of the nodes are proportional to the number of their connections (degree). Hubs with the most connections are indicated by asterisks. Betweenness centrality is considered as one of the most important parameters for the identification of network hubs.

The MCI group had a significantly increased maximum MST degree [*F*(1, 34) = 5.69, *p* = 0.02], degree divergence [*F*(1, 34) = 6.12, *p* = 0.02], and maximum betweenness centrality [*F*(1, 34) = 7.37, *p* = 0.01] (Figure 6) compared to the control group. Furthermore, memory load had a significant modulatory effect on betweenness centrality [*F*(2, 34) = 3.53, *p* = 0.04], indicating a significantly increased BC in the medium memory load condition compared to the low memory load condition (*t* = 2.6, *df* = 34, *p* = 0.01). Leaf fraction and tree hierarchy did not differ significantly in the two groups. Group-average MSTs of the two study groups (average of all levels of memory load) are shown in Figure 6, represented in sensor space. The central hub (the node with the most connections) was the right temporal electrode T8 in both study groups. Detailed results of the MST analysis are summarized in Supplementary Table 2.

### Minimum Spanning Tree Parameters in the Beta Band

The network analysis (calculated over all memory load conditions) indicated that the MCI group had a significantly decreased MST diameter compared to the control group [*F*(1, 34) = 4.58, *p* = 0.04]. Meanwhile, a decreased eccentricity was observed in the MCI group [*F*(1, 34) = 5.62, *p* = 0.02].

The MCI group had a significantly increased maximum MST degree [*F*(1, 34) = 7.55, *p* = 0.01], degree divergence [*F*(1, 34) = 7.15, *p* = 0.01], and maximum betweenness centrality [*F*(1, 34) = 6.95, *p* = 0.01] (Figure 6) compared to the control group. However, age also had a significant mean effect on maximum MST degree and degree divergence [*F*(1, 34) = 5.2, *p* = 0.03 and *F*(1, 34) = 5.44, *p* = 0.03, respectively]. There was no significant difference in leaf fraction and tree hierarchy. Group-average MSTs of the two study groups (average of all levels of memory load) are shown in Figure 6, represented in sensor space. The central hub (the node with the most connections) in the control group was the right temporal electrode T8, while in the MCI group it was the left frontal-temporal electrode FT7. Detailed results of the MST analysis are summarized in Supplementary Table 2.

## Discussion

In this study, we aimed to examine functional connectivity and network structure during memory maintenance in MCI patients and healthy controls.

We used the orthogonalized Amplitude Envelope Correction (AEC-c) for the measurement of functional connectivity, which corrects to the effect of volume conduction, is independent of relative power (Briels et al., 2020), gives reliable estimates of the underlying network topology (Lai et al., 2018), and has been found the most sensitive measure of functional connectivity in the alpha and beta frequency bands (Hipp et al., 2012). Moreover, the AEC-c produced the most reproducible and valid results in AD compared with other measures of functional connectivity, such as mean global coherence (Coh), imaginary coherence (iCoh), phase locking value (PLV), phase lag index (PLI), weighted PLI (wPLI), and the AEC without leakage-correction (Briels et al., 2020).

Alpha and beta-band oscillatory synchrony play an important role in cognitive tasks by mediating top-down directed influences on task-relevant cortical areas (Fries, 2015). Furthermore, alpha synchronization is instrumental in controlling the flow of information especially in the thalamo-cortical and cortico-cortical networks and in the timing of working memory-related processing by the modulation of neural excitability (Klimesch, 1999, 2012; Pfurtscheller and Lopes da Silva, 1999; Palva and Palva, 2011; Wang et al., 2014; Miraglia et al., 2016; Wianda and Ross, 2019) while beta oscillations have been linked to the active maintenance of newly acquired information for further task requirements (Onton et al., 2005; Deiber et al., 2007; Missonnier et al., 2007; Chen and Huang, 2015; Fodor et al., 2018) and to the facilitation of long-range connections in cortical networks (Kopell et al., 2000; Varela et al., 2001; Engel and Fries, 2010; Benchenane et al., 2011; Donner and Siegel, 2011; Kilavik et al., 2013), especially during attentional and memory processes (Benchenane et al., 2011) and endogenous content reactivation (Spitzer and Haegens, 2017). However, many of these previous studies measured functional connectivity by amplitude correlation, whose exact relation to the correlation of amplitude envelopes (which has been used as the measure of connectivity in the present study) and the exact mechanism underlying communication by amplitude envelopes are still not fully understood.

According to our results, memory load modulated the mean functional connectivity in the alpha band, but it had a different modulatory effect in the two study groups. In the control group, increasing task difficulty enhanced the mean functional connectivity. In contrast to that, after an initial increase of the mean AEC-c in the medium memory load condition the MCI group showed significantly diminished functional connectivity in the high memory load condition. The control group showed a similar memory load-related increase in the mean AEC-c in the beta band, while in the MCI group this modulation was absent.

Cognitive load-related increase of alpha and beta band functional connectivity is in line with previous studies which observed the same phenomenon in healthy subjects as well as in MCI and AD patients in alpha and beta band (Pijnenburg et al., 2004; Palva et al., 2010; Wianda and Ross, 2019) and in broadband (Jiang and Zheng, 2006). However, other studies observed the diminishment of task-related increase of coherence of alpha oscillations in AD patients in the dementia phase (based on clinical diagnosis) (Hidasi et al., 2007).

Our results suggest that the AEC-c is a sensitive measure that can follow the modulation of functional connectivity by cognitive demand, especially in the alpha frequency band, which has been associated with attentional functions (Palva et al., 2010; Sato et al., 2018; Marzetti et al., 2019).

We observed that the initial increase of alpha-band functional connectivity in the medium memory load condition was followed by a decrease in the high memory load condition in the MCI group. As former studies linked the increase of alpha-band functional connectivity to enhanced cognitive demand (Pijnenburg et al., 2004; Palva et al., 2010; Wianda and Ross, 2019), and we found a similar modulation in the control group, we hypothesized that the initial increase of alpha connectivity in the MCI group in the medium memory load condition indicates the increased utilization of working memory. However, due to the limited cognitive reserve, MCI patients are unable to act likewise in the high memory load condition as task difficulty exceeds their cognitive capacity. Therefore, the reduction of alpha functional connectivity in the high memory load condition might indicate the reduced cognitive reserve and the impairment of working memory maintenance in MCI, although this was not reflected by a decrease in task performance.

In the beta band, while increasing memory load enhanced functional connectivity in the control group, the MCI group did not show memory load-related modulation. This might indicate a more extensive failure of working memory maintenance in MCI in the beta frequency band. Another possible explanation might be the general “slowing” of EEG in MCI (Dauwels et al., 2010a), namely that task-related dynamics of higher frequency bands got shifted toward lower frequency bands in MCI. The study group did not have a significant modulatory effect on the mean (whole head) AEC-c, which is in line with a previous study on alpha coherence during a working memory task which did not find significant differences between aMCI patients and healthy older adults (van der Hiele et al., 2007b). However, in MCI patients some studies found increased alpha and beta synchronization (Pijnenburg et al., 2004; Jiang, 2005; Jiang and Zheng, 2006), which has been attributed to compensatory mechanisms (Bajo et al., 2010; Dauwels et al., 2010b).

The relatively small sample size of our study may be a factor contributing to the observed lack of between-group differences in mean functional connectivity. A different potential explanation might be that MCI is characterized by increased and decreased functional connectivity in different cortical regions simultaneously (Lopez et al., 2017), and therefore during averaging these changes might be smoothed out. Moreover, most of the previous studies assessed eyes-closed resting-state recordings, while we analyzed eyes-open task-related EEG, which might be another influencing factor.

Alpha and beta AEC-c showed a significant positive correlation with the size of medial temporal lobe structures and a significant negative correlation with the mean diffusivity (MD, a scalar measure of overall water diffusion) of the right hippocampal cingulum in the entire sample. MD increases in the presence of tissue damage and is typically used to assess the microstructural integrity of gray matter (Stebbins and Murphy, 2009). Elevation of hippocampal, parahippocampal, and temporal lobe MD are considered as important early markers of neuronal loss and disruption of myelin sheaths in MCI and AD (Kantarci et al., 2001, 2005; Fellgiebel et al., 2004; Ray et al., 2006; Stebbins and Murphy, 2009; Zhang et al., 2014).

Therefore, our results suggest that functional connectivity and specifically the AEC-c in the alpha and beta frequency band can reflect the subtle medial temporal lobe atrophy and the disruption of hippocampal fiber integrity in the earliest stages of cognitive decline. This is also corroborated by the fact, that these correlations were driven by the MCI subjects, who had a more pronounced hippocampal degeneration compared to the control group.

There is some evidence that changes in MD are more typical in MCI whereas as changes in MD and fractional anisotropy (FA, a measure of the directionality of diffusion) are more typical in AD (Rogalski et al., 2009; Stebbins and Murphy, 2009), which might be the reason why hippocampal FA did not show a significant correlation with mean functional connectivity. Altogether, our results are in line with previous DTI studies reporting a correlation between alpha-band functional connectivity and fiber tract integrity reduction in MCI and mild to moderate AD patients (Teipel et al., 2009; Vecchio et al., 2015).

The two study groups did not differ significantly regarding relative alpha and beta power and peak frequency, in contrast to former studies reporting a widespread decrease of alpha activity in the prefrontal, temporal, parietal, and occipital cortices during the n-back task (San-Martin et al., 2021). In the control group, we found a significant increase of alpha power in the high memory load compared to the low memory load condition in line with former studies (Jensen et al., 2002; Tuladhar et al., 2007; Palva et al., 2011), while this modulatory effect was absent in the MCI group. In the beta band, we did not detect a memory load-related modulation. However, in contrast to the functional connectivity results, the outcome of the power spectrum analysis did not show a load-related modulation of the MCI group in the alpha band (an initial increase followed by a decrease parallel with the enhancement of cognitive load). Therefore, we conclude that the detected differences in functional connectivity are not entirely the consequence of differences of spectral properties, although we cannot rule out the possibility that it might have an influence on the results, especially in the alpha frequency band, which might be a potential limitation.

We performed the network analysis by applying the MST approach, which provides an unbiased reconstruction of the critical backbone of the original network (Stam, 2014; Stam et al., 2014; Tewarie et al., 2015; Wang et al., 2018; Musaeus et al., 2019), and can capture the subtle changes of network topology in MCI more sensitively than traditional graph theoretical measures (Lopez et al., 2017).

We found a decreased MST diameter and eccentricity and increased maximum degree, degree divergence, and maximum betweenness centrality in the MCI group, suggesting a more centralized and integrated network topology compared to the control subjects both in the alpha as well as in the beta frequency band. Our results are in line with former studies, which reported increased BC values and node degree in MCI and AD patients (Engels et al., 2015; Lopez et al., 2017).

The central hub (the node with the most connections) of the group-averaged MST network was the temporal electrode T8 in the alpha band in both study groups and in the beta band in the control group, while it was the left frontal-temporal electrode FT7 in the beta-band MST of the MCI group. The right superior temporal gyrus has been previously identified as an important hub region during working memory maintenance (Park et al., 2011) based on cross-frequency power correlations, however, as our analysis was performed on sensor-space data, we are not able to make precise assumptions about the exact spatial locations of the nodes of the networks. We found a left and slight frontal shift of hub location in the MCI group in the beta frequency band. Interestingly, a frontal shift of hub location (center of mass of BC) was observed with increasing disease severity in AD patients and has been attributed to the earlier impact of the disease pathology on the posterior regions (Engels et al., 2015).

Previous studies interpreted the global network disturbances in MCI and AD by the “hub overload and failure” framework, which states that the initial disturbance of nodes leads to the abnormal rerouting of the information flow in the network to hub nodes with higher centrality leading to an increase of traffic load, and eventually to an overload and subsequent failure of these hub nodes. This stage might also coincide with the initial ascending phase in early MCI of the inverted U shape course of hub activity (de Haan et al., 2012). This initial increase of hub activity and the transition to a more integrated network topology might be part of a compensatory mechanism, but it might as well be a part of the degeneration process itself due to the early impairment of inhibitory neurons (disinhibition) (de Haan et al., 2012). Subsequently, in the chronic “hub failure” phase, these overloaded hubs break down and the rerouting is constrained locally to nodes with a lower level in the hierarchy in the remaining part of the network. This stage also corresponds to the descending phase of the trajectory of hub activity in late MCI and AD (de Haan et al., 2012). This will eventually lead to the disturbance of the modular system of the network (Stam, 2014).

The global network topology reflects this by an initial increase of centralization and a shift from local to global processing followed by a decrease of centrality (Stam, 2014). This transition has been confirmed by fMRI as well, where the MST of MCI patients showed a more star-like topology, while the MST of AD patients deviated toward a more line-like topology compared to healthy controls (Wang et al., 2018).

Our results suggest that brain networks of MCI patients show a transient shift to a more centralized, star-like topology to compensate for the initial impairments in accordance with the “hub overload” stage, and complement former EEG studies, which reported the deviation of the network topology from the optimal small-world architecture to a more random type configuration (Wei et al., 2015) and the shifting of the MST toward a more decentralized, line-like structure of AD patients in the “hub failure” stage during resting state (Yu et al., 2016; Peraza et al., 2018; Das and Puthankattil, 2020) and cognitive tasks (Das and Puthankattil, 2020).

Interestingly, while functional connectivity sensitively reflected changes in cognitive demand, MST network measures did not show significant memory load-related modulation except for maximum BC in the alpha band. This suggests that the AEC-c might be a more state-like attribute, which reflects cognitive demand, while MST network parameters are more trait-like characteristics of MCI and are less dependent on the actual cognitive state.

## Limitations

The present study was limited by the small sample size and a slight age difference between groups, therefore statistical tests were corrected for age as a covariate. However, age had a significant effect on some of the network parameters, which limits the generalizability of our results. Moreover, the PAL test did not have an equal number of trials in the different difficulty levels, which might have influenced the signal-to-noise ratio of the EEG analysis.

Furthermore, we analyzed global functional connectivity to assess robust differences that could be considered as potential biomarkers of cognitive decline. However, a regional analysis focusing especially on the connectivity of the working memory network [prefrontal cortex, the parietal and temporal lobe, and task-specific posterior areas (Ranganath, 2006; Campo and Poch, 2012)] could have provided a more detailed picture of the exact topological distribution of MCI-related differences.

Moreover, in this study, we did not analyze functional connectivity in the theta-band, as the AEC-c produces less reliable and reproducible results in the theta band in contrast to the alpha and beta-band and therefore, it has been suggested that for the assessment of theta-band functional connectivity phase-based measures (PLI) should be used instead of amplitude-based measures (Briels et al., 2020). However, this might be a potential limitation since frontal midline theta activity is an important marker of working memory processing (Jensen and Tesche, 2002; Griesmayr et al., 2010; Sauseng et al., 2010; Kardos et al., 2014).

Furthermore, we performed a scalp-level EEG analysis, which does not allow inferences in terms of underlying neuroanatomy as the location of EEG channels do not relate trivially to the location of the underlying sources, which is a further limitation. It has been suggested, that results derived from scalp-level EEG network should be interpreted cautiously, however, the AEC-c may allow for more reliable estimates of the underlying global network organization compared to metrics that do not correct for the effect of volume conduction (Lai et al., 2018).

Furthermore, while the diagnosis of MCI patients was based on a detailed clinical examination, cerebrospinal fluid biomarkers were not available during the diagnostic procedure. Therefore, AD as the underlying cause of the cognitive disturbance could not be fully proven. Finally, follow-up data is not yet available to examine the predictive value of functional connectivity and network structure in the conversion rate to dementia. Our study provides a cross-sectional view of the changes in functional connectivity and network topology during working memory maintenance in MCI, although further studies in AD biomarker-proven subjects and applying similar paradigms are required to verify our results.

## Conclusion

Our results suggest that the AEC-c sensitively reflects cognitive load-related modulation and impairment of memory retention in MCI. Moreover, alpha and beta-band AEC-c showed a significant correlation with the size of medial temporal lobe structures and with the mean diffusivity of the right hippocampal cingulum, therefore, the AEC-c can reflect subtle medial temporal lobe atrophy and the disruption of hippocampal fiber integrity in the earliest stages of cognitive decline.

Furthermore, the MST network topology of the MCI group showed a more centralized and integrated configuration compared to the healthy control subject, which is in line with the “hub overload and failure” framework, and might be part of a compensatory mechanism or a consequence of neural disinhibition.

Therefore, the assessment of EEG functional connectivity and network structure in the alpha and beta frequency range may provide a useful complementary diagnostic tool for the early detection of cognitive impairment and might be a step toward establishing functional biomarkers (Sharma et al., 2019). However, future research applying similar paradigms is required to further develop and confirm these initial findings by using follow-up data to determine the predictive value of functional connectivity measures and network parameters for future conversion to dementia.

## Data Availability Statement

The raw data supporting the conclusions of this article will be made available by the authors, without undue reservation.

## Ethics Statement

The studies involving human participants were reviewed and approved by the National Scientific and Ethical Committee, Budapest, Hungary. The patients/participants provided their written informed consent to participate in this study.

## Author Contributions

GC designed the study, wrote the protocol, and contributed to the writing of all sections. ZF participated in the execution of measurements, managed the literature searches, undertook the statistical analysis, prepared the figures, and wrote the first draft of the manuscript. AH participated in the execution of measurements and contributed to the writing of all sections. ZH, AG, and CS contributed to the conceptualization of the study and the writing of all sections. All authors reviewed the manuscript.

## Conflict of Interest

The authors declare that the research was conducted in the absence of any commercial or financial relationships that could be construed as a potential conflict of interest.

## Publisher’s Note

All claims expressed in this article are solely those of the authors and do not necessarily represent those of their affiliated organizations, or those of the publisher, the editors and the reviewers. Any product that may be evaluated in this article, or claim that may be made by its manufacturer, is not guaranteed or endorsed by the publisher.

## Footnotes

1
http://surfer.nmr.mgh.harvard.edu/

## Supplementary Material

Supplementary Figure 1 Distribution of mean relative power in the alpha and beta frequency band and peak frequency in the study groups.

Supplementary Table 1 Correlations between the temporal lobe structures and mean functional connectivity stratified by diagnosis.

Supplementary Table 2 MST metrics in the alpha and beta frequency band.
