# Supplementary figures and images for: EEG Alpha and Beta Band Functional Connectivity and Network Structure Mark Hub Overload in Mild Cognitive Impairment During Memory Maintenance
Source: Front Aging Neurosci. 2021 Oct 7;13:680200. doi: 10.3389/fnagi.2021.680200 (PMC8529331; doi:10.3389/fnagi.2021.680200)

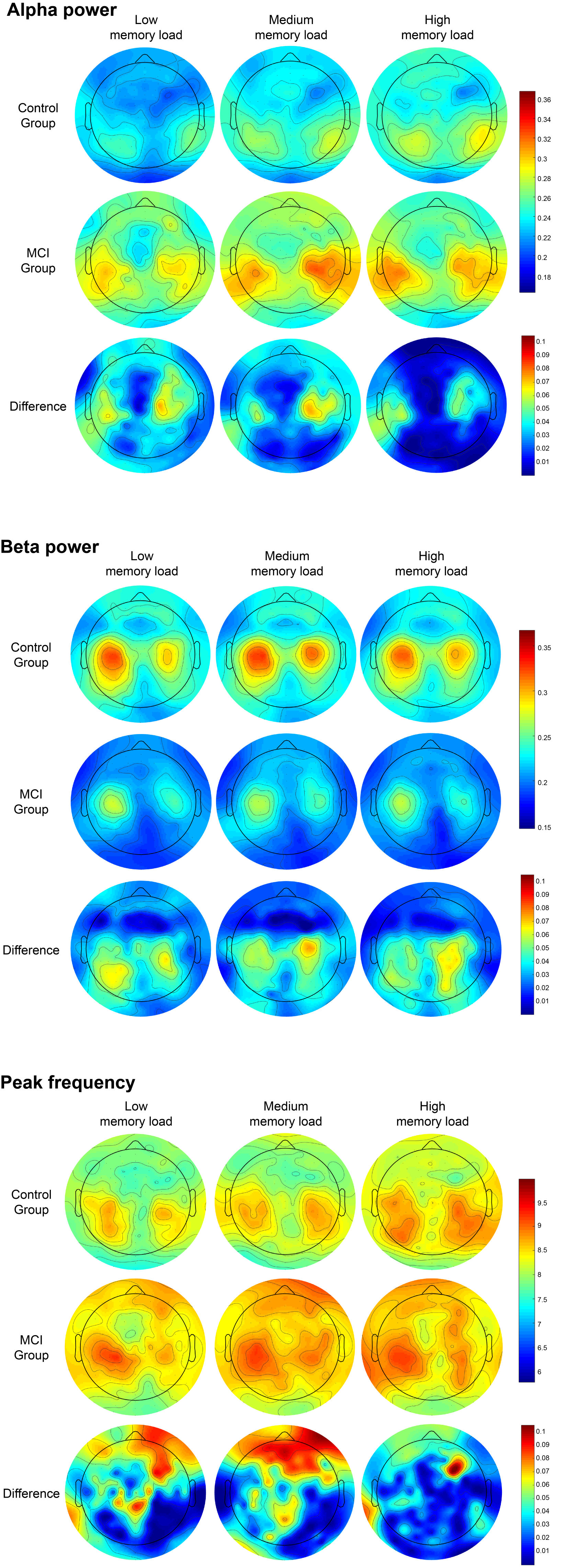

Supplement: Supplementary Figure 1 — Distribution of mean relative power in the alpha and beta frequency band and peak frequency in the study groups. [file Image_1.TIFF]
