# Supplementary material for: EEG Alpha and Beta Band Functional Connectivity and Network Structure Mark Hub Overload in Mild Cognitive Impairment During Memory Maintenance
Source: Front Aging Neurosci. 2021 Oct 7;13:680200. doi: 10.3389/fnagi.2021.680200 (PMC8529331; doi:10.3389/fnagi.2021.680200)
Supplement: Supplementary Table 1 — Correlations between the temporal lobe structures and mean functional connectivity stratified by diagnosis. [file Table_1.DOCX]

**Supplementary Table 1. Correlations between the temporal lobe structures and mean functional connectivity stratified by diagnosis**

|  | **Mean functional connectivity (AEC-c)** | | | |
| --- | --- | --- | --- | --- |
| **Structural MRI results (n = 26)** | **Alpha band** | | **Beta band** | |
| **Volume (mm^3^)** |  | |  | |
|  | **Control (n = 13)** | **MCI (n = 13)** | **Control (n = 13)** | **MCI (n = 13)** |
| Hippocampus *Spearman r (p-value)* | 0.18 (0.55) | 0.44 (0.13) | 0.29 (0.36) | **0.72 (0.008)** |
| **Cortical thickness (mm)** |  | |  | |
|  | **Control (n = 13)** | **MCI (n = 13)** | **Control (n = 13)** | **MCI (n = 13)** |
| Entorhinal cortex *Spearman r (p-value)* | -0.32 (0.29) | 0.32 (0.29) | -0.0003 (0.99) | **0.70 (0.01)** |
| Parahippocampal gyrus *Spearman r (p-value)* | 0.16 (0.60) | 0.55 (0.05) | 0.31 (0.33) | **0.60 (0.04)** |
| **Diffusion weighted MRI results (n = 27)** | | | | |
| **Mean diffusivity (MD)** |  |  |  |  |
|  | **Control (n = 17)** | **MCI (n = 10)** | **Control (n = 17)** | **MCI (n = 10)** |
| Right cingulum – Hippocampus *Spearman r (p-value)* | -0.05 (0.84) | **-0.83 (0.003)** | -0.36 (0.16) | **-0.67 (0.03)** |
| Left cingulum – Hippocampus *Spearman r (p-value)* | 0.03 (0.92) | -0.35 (0.33) | -0.11 (0.67) | -0.35 (0.33) |
| **Fractional anisotropy (FA)** |  |  |  |  |
|  | **Control (n = 17)** | **MCI (n = 10)** | **Control (n = 17)** | **MCI (n = 10)** |
| Right cingulum – Hippocampus *Spearman r (p-value)* | -0.16 (0.55) | 0.49 (0.15) | -0.002 (0.99) | 0.60 (0.07) |
| Left cingulum – Hippocampus *Spearman r (p-value)* | -0.04 (0.87) | **0.66 (0.04)** | 0.09 (0.72) | 0.38 (0.28) |
