# Supplementary material for: EEG Alpha and Beta Band Functional Connectivity and Network Structure Mark Hub Overload in Mild Cognitive Impairment During Memory Maintenance
Source: Front Aging Neurosci. 2021 Oct 7;13:680200. doi: 10.3389/fnagi.2021.680200 (PMC8529331; doi:10.3389/fnagi.2021.680200)
Supplement: Supplementary Table 2 — MST metrics in the alpha and beta frequency band. [file Table_2.DOCX]

**Supplementary Table 2. MST metrics in the alpha and beta frequency band**

|  |  | **Low memory load** | | **Medium memory load** | | **High memory load** | |
| --- | --- | --- | --- | --- | --- | --- | --- |
|  | **MST parameter** | **Control (n = 20)** | **MCI (n = 17)** | **Control (n = 20)** | **MCI (n = 17)** | **Control (n = 20)** | **MCI (n = 17)** |
|  |  | **Mean (SD)** | **Mean (SD)** | **Mean (SD)** | **Mean (SD)** | **Mean (SD)** | **Mean (SD)** |
| **Alpha frequency**  **band** | **Degree** | 0.131 (0.01) | 0.159 (0.04) | 0.141 (0.01) | 0.150 (0.03) | 0.139 (0.02) | 0.150 (0.03) |
|  | **Leaf** | 0.653 (0.02) | 0.668 (0.02) | 0.658 (0.02) | 0.669 (0.03) | 0.660 (0.02) | 0.665 (0.02) |
|  | **Diameter** | 0.140 (0.01) | 0.134 (0.01) | 0.136 (0.01) | 0.133 (0.01) | 0.136 (0.01) | 0.134 (0.01) |
|  | **eccentricity** | 0.107 (0.004) | 0.103 (0.01) | 0.105 (0.01) | 0.103 (0.01) | 0.104 (0.01) | 0.103 (0.01) |
|  | **BC** | 0.695 (0.01) | 0.706 (0.02) | 0.704 (0.02) | 0.712 (0.02) | 0.699 (0.02) | 0.710 (0.02) |
|  | **Th** | 0.474 (0.01) | 0.478 (0.01) | 0.472 (0.02) | 0.474 (0.02) | 0.476 (0.02) | 0.473 (0.01) |
|  | **Kappa** | 4.510 (0.29) | 5.270 (1.04) | 4.709 (0.36) | 5.049 (0.92) | 4.742 (0.61) | 5.050 (0.85) |
|  |  | **Low memory load** | | **Medium memory load** | | **High memory load** | |
|  | **MST parameter** | **Control (n = 20)** | **MCI (n = 17)** | **Control (n = 20)** | **MCI (n = 17)** | **Control (n = 20)** | **MCI (n = 17)** |
|  |  | **Mean (SD)** | **Mean (SD)** | **Mean (SD)** | **Mean (SD)** | **Mean (SD)** | **Mean (SD)** |
| **Beta frequency**  **band** | **Degree** | 0.133 (0.02) | 0.155 (0.03) | 0.136 (0.02) | 0.156 (0.04) | 0.139 (0.02) | 0.153 (0.05) |
|  | **Leaf** | 0.660 (0.02) | 0.668 (0.02) | 0.663 (0.02) | 0.671 (0.02) | 0.666 (0.02) | 0.667 (0.03) |
|  | **Diameter** | 0.134 (0.01) | 0.129 (0.01) | 0.133 (0.01) | 0.129 (0.01) | 0.130 (0.01) | 0.129 (0.01) |
|  | **Eccentricity** | 0.104 (0.01) | 0.099 (0.01) | 0.103 (0.01) | 0.099 (0.01) | 0.100 (0.01) | 0.099 (0.01) |
|  | **BC** | 0.702 (0.01) | 0.714 (0.02) | 0.699 (0.01) | 0.713 (0.02) | 0.710 (0.02) | 0.717 (0.03) |
|  | **Th** | 0.474 (0.01) | 0.472 (0.01) | 0.478 (0.01) | 0.475 (0.02) | 0.473 (0.02) | 0.469 (0.02) |
|  | **Kappa** | 4.594 (0.42) | 5.166 (0.93) | 4.652 (0.52) | 5.197 (0.96) | 4.775 (0.44) | 5.186 (1.40) |

*MCI: Mild cognitive impairment, MST: Minimum Spanning Tree, BC: betweenness centrality, Th: tree hierarchy, Kappa: degree divergence*
